# Simulation of PLC Effect Using Regularized Large-Strain Elasto-Plasticity

**DOI:** 10.3390/ma15124327

**Published:** 2022-06-18

**Authors:** Marzena Mucha, Balbina Wcisło, Jerzy Pamin

**Affiliations:** Chair for Computational Engineering, Faculty of Civil Engineering, Cracow University of Technology, Warszawska 24, 31-155 Cracow, Poland; balbina.wcislo@pk.edu.pl (B.W.); jerzy.pamin@pk.edu.pl (J.P.)

**Keywords:** PLC effect, visco-plasticity, thermo-mechanical coupling, gradient enhancement, FEM

## Abstract

The purpose of this paper is to develop a constitutive description and to numerically simulate a propagating instability phenomenon called the Portevin–Le Chatelier (PLC) effect, which is observed for metallic materials. It manifests itself by moving plastic shear bands in the sample and serrations in the stress–strain diagram. In this paper, the PLC is modeled by geometrically non-linear thermo-visco-plasticity with the hardening function of the Estrin–McCormick type to reproduce a serrated response. To regularize softening, which in this model comes from thermal, geometrical and strain-rate effects, the viscosity and heat conductivity are incorporated. Plasticity description can additionally include degradation of the yield strength, and then the model is enhanced by higher-order gradients. Simulations are performed using AceGen/FEM. Two tensioned specimens are tested: a rod and a dog-bone sample. The first specimen is used for general verification. The results obtained for the second specimen are compared with the experimental results. Studies for different values of model parameters are performed. The results of the simulations are in good agreement with the experimental outcome and the sensitivity to model parameters is in line with the expectations for the pre-peak regime. In the presented tests, the gradient enhancement does not significantly influence the results.

## 1. Introduction

The Portevin–Le Chatelier (PLC) effect is an instability phenomenon that manifests itself in bands of localized plastic strain rate, propagating along a stressed specimen. It is related to stress jumps (serrations) in the load-displacement diagram, which represents a specimen response under tension or shear. The source of this behaviour lies in the microstructure evolution, in particular at the level of dislocation motion. It is specifically exhibited by steel and aluminium alloys and occurs for a certain range of strain rates and temperatures. The PLC can reduce ductility and formability of alloys; hence, its analysis is of both theoretical and practical importance.

The plastic flow in metals and alloys can be explained by nucleation and motion of dislocations. The motion can be blocked by other dislocations, causing dislocation pile-up which can be unlocked by a sufficiently large strain. In solid solutions, dislocations can also be stopped by diffused solute atoms. The dislocation pinning by the solutes, repeatedly followed by unpinning, produces instabilities in the plastic flow. They occur as serrations in the stress–strain diagram, related to the motion (or repeated occurrence and vanishing) of localized strain-rate bands along a stressed specimen.

The micro-structural phenomenon responsible for the PLC effect is so-called Dynamic Strain Aging (DSA) [1,2]. As explained above, DSA is related to dynamic interactions between the motion of mobile dislocations and the diffusion of solute atoms. The recurring decrease in the concentration of solute atoms at temporarily arrested dislocations is represented by a reduction in solute contribution to the flow stress.

The PLC effect was first reported by [3]. Experimental studies and analytical models of propagative instabilities, in particular of Lueders bands and the Portevin–Le Chatelier effect, were discussed in [4]. An extensive numerical study of the phenomena using small-strain isothermal elasto-plasticity models is provided in [5,6]. Finite element models of the PLC effect are analyzed there in the context of regularized dynamics. An overview of the experiments showing the PLC phenomenon, including a classification of its types, is presented in [7]. Experimental analysis and modeling of the three types is performed in [8]. A review of modeling options for the phenomenon is provided in [9].

The PLC effect can be described, among others, by the Estrin–McCormick model [10,11,12,13]. In [10], the model is derived; in [11,12], it is applied in small-strain FE simulations of the phenomenon. In [13], the model is implemented within a large strain model of elasto-plasticity, including parameter identification for an aluminium alloy based on experiments on tensile specimens under loading with different rates.

In recent years, several scientists have considered the phenomenon in their theoretical, experimental and numerical studies. In particular, steel specimens were examined in [14,15,16,17,18] (the last paper covers an experimental study of the PLC phenomenon in high-strength steel) and aluminium alloys in a larger number of works; for instance, [13,19,20,21,22].

The majority of specimens used in the studies were rectangular or dog-bone shape tensile plates (see [8,13,15,19,20,23]), sometimes notched (e.g., [17]), and tensile rods with circular cross-section (see for instance [23]). Some studies concerned shear specimens [21,24,25]. Several papers compare the experimental response (in some cases monitored using the DIC technique) with simulation results; see, for instance [17,19,22,26,27,28]. The final failure, involving necking and fracture, was examined in [15]. Moreover, in some papers, the influence of loading/strain rate has been examined, see [8,13,20,24,29].

A few studies considered temperature dependence of the PLC effect. These were [20,25,30,31]. The simulation of the temperature-dependent process zone at the crack tip was in the focus of [14]. Finally, it is mentioned that a constitutive model of discontinuous plastic flow for materials deformed at cryogenic temperatures was developed in [32] and further considered in [33]. In fact, these papers and the present one belong to the broad field of research on thermo-mechanics of heterogeneous/composite materials and structures. The thermo-mechanical couplings are constantly a subject of intensive scientific activity; see, for instance [34,35,36,37,38,39,40,41].

In the present paper, the PLC effect is simulated using a formulation of geometrically non-linear thermo-plasticity developed in [42,43]. The model includes full thermo-mechanical coupling involving thermal expansion, plastic heating, thermal softening in the plasticity function, and Fourier’s law in the deformed configuration. Following [44,45], the thermo-elastic coupling is neglected in the energy balance because it is relatively insignificant for the metallic materials under consideration.

The plasticity description is based on [46] and includes the Huber–Mises–Hencky yield criterion. This model was recently extended to visco-plasticity and was employed by the authors to simulate Lueders bands in [47]. The rate-dependent hardening function of the Estrin–McCormick type, described in [13], is used to simulate DSA and the serrations induced by strain-rate softening, but it is enhanced with temperature dependence of the characteristic time of solute diffusion.

The models are implemented in an AceGen code generator developed in [48] within Wolfram Mathematica. One of the aims of the research is to examine the influence of temperature and strain rates on the PLC phenomenon, and the importance of regularization in the models involving recurring strain-rate-softening phases. It is stressed here that the stabilizing effect is provided by viscosity and heat conduction, but can also be provided by a gradient enhancement.

The paper is organized as follows. In Section 2, the theory of large strain thermo-visco-plasticity is summarized and then extended to include the Estrin–McCormick component of the yield strength, which makes it possible to simulate the PLC effect. Interest is limited to tension and moderate temperatures (20–200 degrees Celsius). A gradient enhancement of the model is optionally incorporated to regularize the softening involved in the model. In Section 3 the coupled balance equations are presented in local and weak forms, and then the implementation of the model in AceGen/FEM for Mathematica is briefly discussed. In Section 4, the simulation results are presented. First, a one-dimensional rod model is considered and detailed response is shown for one serration. Then, a series of simulations for a dog-bone specimen under tension is presented. The computed model is based on the experimental research on aluminium dog-bone-type specimens, presented in [49]. Comparisons with laboratory test results are made, and some parametric studies are performed. Finally, in Section 5 some conclusions are drawn and directions of future work are proposed.

## 2. Brief Description of Constitutive Models

### 2.1. Thermo-Visco-Plasticity

The material models used in this paper for the simulation of the PLC-type instabilities are described below. They are based on the large-strain description of elasto-plasticity [13,45,50,51].

The starting point of the formulation is standard. We consider a continuous deformable body and its material is assumed to be initially isotropic. Vector X identifies the reference location of a body particle at time t=0 and in initial temperature $T_{0}$ ($T_{0}$ is assumed to be the reference temperature for a strain-free state), vector x points to the current position of the particle at time *t* and in temperature *T*. The motion of the body is described by function x=φ(X,t,T). The classical definition of the deformation gradient F is recalled:

(1) $$F=\frac{\partial \varphi (X,t,T)}{\partial X}.$$

A multiplicative decomposition of F into mechanical and thermal parts denoted by $F^{m}$ and $F^{\theta }$, respectively, is performed [45,52]. In turn, $F^{m}$ is decomposed into elastic and plastic factors $F^{e}$ and $F^{p}$ (see [53,54,55])

(2) $$F=F^{m}F^{\theta }=F^{e}F^{p}F^{\theta }.$$

The thermal factor $F^{\theta }$ is assumed to be purely volumetric and defined as

(3) $$F^{\theta }={\left(J^{\theta }\right)}^{1/3}I\;,\;J^{\theta }=\det\left(F^{\theta }\right),$$

where I is the second-order identity tensor. The volumetric deformation caused by the temperature change $T-T_{0}$ is represented by [55]

(4) $$J^{\theta }=\exp\left[3\alpha_{T}\left(T-T_{0}\right)\right],$$

where $\alpha_{T}$ is the coefficient of linear thermal expansion. Based on Equations (2) and (4) the mechanical part of the deformation gradient is derived as

(5) $$F^{m}=\exp\left[-\alpha_{T}\left(T-T_{0}\right)\right]F.$$

For the classical thermo-plasticity theory, the Helmholtz potential calculated per unit volume in the reference configuration is decomposed into elastic, plastic, and purely thermal components (see [45,50])

(6) $$\psi \left(b^{e},\alpha ,T\right)=\psi^{e}\left(b^{e}\right)+\psi^{p}\left(\alpha \right)+\psi^{\theta }\left(T\right).$$

The following definitions of the potential parts are employed.

(7) $$\psi^{e}\left(b^{e}\right)=\frac{1}{2}G\left[\mathrm{tr}(\det{\left(b^{e}\right)}^{-1/3}b^{e})-3\right]+\frac{1}{2}K\ln{\left(J^{e}\right)}^{2},$$

(8) $$\psi^{p}\left(\alpha \right)=\left(\sigma_{yf}-\sigma_{y0}\right)\left[\alpha +\frac{\exp(-\delta \alpha )}{\delta }\right],$$

(9) $$\psi^{\theta }\left(T\right)=c\left[\left(T-T_{0}\right)-T\;\ln\left(\frac{T}{T_{0}}\right)\right].$$

In Equation (7), *G* and *K* are shear and bulk elastic moduli, $b^{e}=F^{e}{\left(F^{e}\right)}^{T}$ is the elastic left Cauchy–Green tensor, and $J^{e}=\det\left(F^{e}\right)$.

The second component of the free energy represents plastic hardening and is assumed in the form relevant for saturation-type hardening with a scalar equivalent plastic strain α, $\sigma_{y0}$ is an initial yield strength, $\sigma_{yf}$ is a final yield strength, and δ is a saturation constant. In general, $\psi^{p}$ depends on adopted hardening specification. Moreover, in Equation (9), *c* is the heat capacity per unit of volume. According to [46] it can be defined as $c=-T\frac{\partial^{2}\psi }{\partial T^{2}}$ and therefore for the adopted form of free energy, *c* is constant.

The Kirchhoff stress tensor τ and hardening function h(α) are derived from the free energy potential

(10) $$\tau =2\frac{\partial \psi }{\partial b^{e}}b^{e},\;h=\frac{\partial \psi }{\partial \alpha }.$$

The constitutive relation for heat conduction is the classical Fourier law for isotropic materials. It is formulated according to [46] using the Kirchhoff heat flux vector q

(11) q=−k∇T,

where *k* is a heat conduction coefficient specified in the reference configuration and ∇T is a spatial gradient of temperature.

Further, the plasticity formulation is specified. The yield function is defined as

(12) $$F_{p}\left(\tau ,\alpha ,\dot{\alpha }\right)=f\left(\tau \right)-\sigma_{y}\left(\alpha ,\dot{\alpha }\right)\leq 0,$$

where f(τ) is the Huber–Mises–Hencky (HMH) stress measure and $\sigma_{y}$ represents the evolving yield strength (flow stress) for the rate-dependent (viscoplastic) model, which is the starting point of the derivation. The viscoplasticy formulation follows the consistency concept, cf. [5]. The particular forms of $\sigma_{y}$ will be discussed in the next section for the Estrin–McCormick visco-plasticity model and a gradient-enhanced version of the model. The following definitions are used

(13) $$f\left(\tau \right)=\sqrt{2J_{2}},$$

(14) $$J_{2}=\frac{1}{2}\tau_{dev}^{2}\cdot I,$$

where $\tau_{dev}$ is deviatoric part of the Kirchhoff stress tensor and I is the second order unit tensor.

The yield function presented in Equation (12) has a general form which can easily be modified to apply another yield criterion. The Huber–Mises–Hencky function is chosen in the work because it describes the behaviour of metals satisfactorily. This form of stress measure is independent of the hydrostatic pressure, which implies the isochoric plastic flow. In this approach, the volumetric-deviatoric split of large-strain measures does not need to be incorporated in the description. It is worth mentioning that the volume of the material can change due to thermal expansion and elastic deformation.

Following [50], the associated flow rule is adopted for the Lie derivative of $b^{e}$

(15) $$-\frac{1}{2}L_{v}b^{e}=\dot{\gamma }\frac{\partial F_{p}}{\partial \tau }b^{e},$$

where $\dot{\gamma }$ denotes the plastic multiplier satisfying the standard Kuhn–Tucker conditions:

(16) $$\dot{\gamma }\geq 0,\;F_{p}\leq 0,\;\dot{\gamma }F_{p}=0.$$

According to [56], the plastic multiplier plays the role of the plastic strain measure according to the relation

(17) $$\dot{\alpha }=\sqrt{2/3}\dot{\gamma }.$$

### 2.2. Estrin–McCormick Model with Optional Gradient Enhancement

The Estrin–McCormick model (further called the McCormick model or the EMC model in brief) is a phenomenological description of DSA based on an internal variable called effective strain aging time $t_{a}$, cf. [10,12,13]. The evolution of $t_{a}$ introduces repeated negative strain-rate dependence, causing the serrations related to nucleating and propagating localization bands.

In this paper, the model is extended to include temperature dependence, cf. [9,25]. The yield function is defined as

(18) $$F_{p}\left(\tau ,\alpha ,\dot{\alpha },t_{a},T\right)=f\left(\tau \right)-\sqrt{2/3}\sigma_{y}\left(\alpha ,\dot{\alpha },t_{a},T\right)\leq 0.$$

It is assumed for simplicity that the standard hardening has a saturation character, viscosity does not depend on temperature, and thermal softening is linear. The yield strength $\sigma_{y}$ depends on equivalent plastic strain α, its rate $\dot{\alpha }$, strain aging time $t_{a}$, and temperature *T*. It has three components related to strain hardening $\sigma_{H}$, strain rate sensitivity $\sigma_{V}$, and dynamic strain aging $\sigma_{B}$:

(19) $$\sigma_{y}\left(\alpha ,\dot{\alpha },t_{a},T\right)=\sigma_{H}\left(\alpha ,T\right)+\sigma_{V}\left(\dot{\alpha }\right)+\sigma_{B}\left(\alpha ,t_{a},T\right).$$

The first component represents the saturation hardening scaled by a thermal softening factor

(20) $$\sigma_{H}\left(\alpha \right)=\left[\sigma_{y0}+\left(\sigma_{yf}-\sigma_{y0}\right)(1-\exp\left(-\delta \alpha \right)\right]\left[1-H_{T}\left(T-T_{0}\right)\right].$$

The part $\left(1-H_{T}\left(T-T_{0}\right)\right)$ corresponds to linear thermal softening, and $H_{T}$ is a thermal softening modulus. Alternative formulae for thermal softening and their discussion in the context of strain localization simulations can be found in [57].

The second component of the yield strength introduces positive strain rate influence (ξ is viscosity parameter)

(21) $$\sigma_{V}\left(\dot{\alpha }\right)=\xi \dot{\alpha }.$$

It is noted that the time derivative of α in the viscous term $\xi \dot{\alpha }$ in Equation (21) is computed using the backward Euler scheme as follows

(22) $$\dot{\alpha }=\frac{\alpha_{n}-\alpha_{n-1}}{\Delta t},$$

where $\alpha_{n}$ and $\alpha_{n-1}$ denote the values of the equivalent plastic strain at the current and previous time moments, respectively, and Δt is a time step.

The third component represents the influence of the DSA according to [13]

(23) $$\sigma_{B}\left(t_{a},\alpha \right)=\sigma_{B0}\left(\alpha \right)\left[1-\exp{\left(-\frac{t_{a}}{t_{0}}\right)}^{n}\right].$$

The formula particularly expresses the solute concentration at temporarily stopped mobile dislocations, which involves negative strain-rate sensitivity. It is driven by the strain aging time $t_{a}$. Moreover, $t_{0}$ is the characteristic time for the solute diffusion, which determines how fast the saturation of hardening component $\sigma_{B}$ is reached. The saturation factor $\sigma_{B0}$ (the maximum value of contribution $\sigma_{B}$) is assumed to depend linearly on the accumulated plastic strain measure α:

(24) $$\sigma_{B0}=\sigma_{B00}+\sigma_{B00}^{\prime }\alpha ,$$

where $\sigma_{B00}$ and $\sigma_{B00}^{\prime }$ are model parameters as well as exponent *n* in Equation (23).

The strain aging time $t_{a}$ is related to a waiting time $t_{w}$ (which a dislocation spends at an obstacle) by the differential equation:

(25) $${\dot{t}}_{a}=1-\frac{t_{a}}{t_{w}},$$

where the waiting time is related to the plastic strain rate $\dot{\alpha }$

(26) $$t_{w}=\frac{\Omega (\alpha )}{\dot{\alpha }},$$

and the plastic strain increment Ω associated with the motion of dislocations between two obstacles (pinned configurations) is also assumed to depend linearly on α:

(27) $$\Omega \left(\alpha \right)=\Omega_{0}+\Omega_{0}^{\prime }\alpha .$$

In the above equation $\Omega_{0}$ and $\Omega_{0}^{\prime }$ are model parameters.

Algorithmically, the evolution of $t_{a}$ depends on the plastic strain increment Δα and can be computed for time increments as follows [13]:

(28) $$t_{a}=\frac{t_{a}^{n}+\Delta t}{1+\frac{\Delta \alpha }{\Omega (\alpha^{n}+\Delta \alpha )}},$$

where the plastic strain rate $\dot{\alpha }$ has been approximated according to Equation (22).

Now, the model depends on temperature in a couple of ways: due to thermal expansion, plastic heating and thermal softening. It is assumed that only the basic mechanical parameters (Young modulus, initial and final yield strength) depend on temperature. Additionally, to examine the sensitivity of the McCormick model to temperature, the following dependence of parameter $t_{0}$ on temperature is assumed:

(29) $$t_{0}\left(T\right)=t_{02}\exp\left(t_{01}T\right).$$

The parameters of this function $t_{01}$ and $t_{02}$ have been determined on the basis of experimental results presented in [25].

It is emphasized that, next to thermal softening assumed in Equation (20) and strain-rate softening present in Equation (23), geometrical softening due to large deformations is also present in the description; see, for instance, [58]. This version of the McCormick model incorporates two regularizing effects, i.e., rate dependence and heat conduction.

In more detailed material modeling, the first component of the yield strength can additionally include a damage-type reduction to represent an increasing porosity of the material related to large strains and leading to fracture. This extension of the model is here based on [51] where a reducing factor exp(−βz) decays from one to zero with increasing material degradation (β is a ductility parameter) and scales the hardening part of the yield strength. In the local version of the model, *z* would be taken equal to α; however, to control the influence of the degradation coefficient on the plastic strain localization process *z* is rather an averaged plastic strain measure, obtained from the following averaging equation [59]

(30) $$z-l^{2}\nabla_{0}^{2}z=\alpha ,$$

in which *l* is an internal length scale and, since so-called Lagrange averaging is employed according to [60], $\nabla_{0}$ is the gradient operator in the material description. Homogeneous natural boundary conditions are assumed for Equation (30).

The yield function then reads

(31) $$F_{p}\left(\tau ,\alpha ,\dot{\alpha },z,t_{a},T\right)=f\left(\tau \right)-\sqrt{2/3}\sigma_{y}\left(\alpha ,\dot{\alpha },z,t_{a},T\right)\leq 0,$$

and the flow stress depends on equivalent plastic strain α, its rate $\dot{\alpha }$, (non-local) degradation parameter *z*, strain aging time $t_{a}$, and temperature *T*

(32) $$\sigma_{y}\left(\alpha ,\dot{\alpha },z,t_{a},T\right)=\sigma_{H}\left(\alpha ,z,T\right)+\sigma_{V}\left(\dot{\alpha }\right)+\sigma_{B}\left(\alpha ,t_{a},T\right),$$

where

(33) $$\sigma_{H}\left(\alpha \right)=\left[(\sigma_{y0}+\left(\sigma_{yf}-\sigma_{y0}\right)\left(1-\exp\left(-\delta \alpha \right)\right)\right]\exp\left(-\beta z\right)\left[1-H_{T}\left(T-T_{0}\right)\right],$$

represents saturation hardening scaled by the degradation coefficient exp(−βz) and by the linear thermal softening factor $1-H_{T}\left(T-T_{0}\right)$. This last version of the model thus incorporates the rate and gradient dependence simultaneously; cf., for instance [61,62].

## 3. Balance Equations

Due to the distinction between the reference and the current configurations in the large strain analysis, the governing equations can be formulated in the material or spatial description; see, for instance, [46,63], respectively. In the described model, spatial quantities are used, but they refer to the volume or surface in the reference configuration; see [64].

The first governing equation for the analyzed coupled problem imposes static equilibrium in the local form

(34) Jdiv(τ/J)=0.

In Equation (34) div(·) is the divergence computed with respect to spatial coordinates and body forces have been neglected. The equilibrium Equation (34) is completed with the boundary conditions for displacement vector u and for traction vector t:

(35) $$\begin{array}{c} u=\hat{u}\;\mathrm{on}\;\partial B_{u},\\ t=\tau \cdot n=\hat{t}\;\mathrm{on}\;\varphi \left(\partial B_{\tau }\right), \end{array}$$

where n is the normal to the body surface.

The second governing equation represents the energy balance written in the temperature form for a non-stationary heat transport, as follows.

(36) $$c\frac{\partial T}{\partial t}-J\mathrm{div}\left(-q/J\right)-R=0.$$

In Equation (36), R is a heat source density per unit of volume. It includes so-called plastic heating, i.e., the source density due to plastic dissipation written in the simple form [45]

(37) $$R=\chi \sigma_{y}\dot{\alpha },$$

where χ denotes a heat dissipation factor [65], assumed to be constant. The energy balance Equation (36) is completed with appropriate boundary conditions:

(38) $$\begin{array}{c} T=\hat{T}\;\mathrm{on}\;\partial B_{T},\\ q\cdot n=\hat{q}\;\mathrm{on}\;\varphi \left(\partial B_{q}\right), \end{array}$$

and an initial condition stating that for t=0 we have $T=T_{0}\;\mathrm{in}\;B$.

For the gradient-enhanced version of the model, the averaging Equation (30) is an additional balance equation. The averaged plastic strain *z* is an additional fundamental unknown, discretized in addition to displacements and temperature, leading to a three-field formulation of the coupled BVP.

The weak forms of the governing equations are the basis for the finite element implementation. Multiplication of Equation (34) by test function δu, integration over the volume of body B and application of the divergence theorem as well as Neumann boundary conditions lead to the weak form of the linear momentum balance

(39) $$\int_{B}\left(\nabla \delta u:\tau \right)dV+\int_{\varphi (\partial B_{\tau })}\delta u\cdot \hat{t}da=0.$$

The weak form of Equation (36) is obtained using the standard procedure and the backward Euler scheme for time integration. As a result, the following integral equation is required to be valid for the current time

(40) $$\int_{B}\left[\delta T\frac{c}{\Delta t}\left(T-T_{n}\right)+k\nabla \delta T\cdot \nabla T-\delta TR\right]dV+\int_{\varphi (\partial B_{q})}\delta T\hat{q}da=0,$$

where $T_{n}$ is the value of temperature at the previous time moment and Δt is the time increment. Finally, the weak form of Equation (30) is written as follows

(41) $$\int_{B}\left[\delta z\left(z-\alpha \right)+l^{2}\nabla_{0}\delta z\cdot \nabla_{0}z\right]dV=0.$$

Equations (39), (40), and optionally (41) are required to be valid for any admissible weighting functions δu, δT and δz, respectively. After the introduction of finite element approximations of the two or three fundamental unknowns according to the Galerkin approach, a set of algebraic equations can be obtained for a monolithic solution algorithm.

## 4. Implementation and Computational Tool

The numerical implementation and testing of the coupled model is performed in Wolfram Mathematica packages AceGen and AceFEM, developed by Korelc [66]. The first package is used to program user-supplied procedures for the finite element method, in particular the tangent and residual subroutine for the Newton-Raphson algorithm and the postprocessing subroutine. The code is prepared in a special meta-language and automatically translated by AceGen. The routine can then be transferred to a chosen finite element environment (e.g., ABAQUS, FEAP), but an integrated FE engine AceFEM can also be used, and this is the case here. AceFEM is equipped with convenient pre- and post-processing tools and perfectly cooperates with AceGen in the computational process.

The main advantage of AceGen, from the researcher’s point of view, is its ability to perform automatic differentiation of symbolic expressions. It is worth emphasising that the material model which is developed to simulate the PLC phenomenon results in a highly non-linear two- (or three-) field problem which is solved using the iterative Newton–Raphson algorithm which requires linearization of the governing equations. This part of the model preparation is very often the most challenging part of the implementation process. The application of automatic differentiation in the AceGen package significantly improves this step. If the residual (with all explicit and nested dependencies between variables) is properly defined, then the tangent matrix components are computed automatically as derivatives of the residual with respect to the unknowns. What is more, the finite element subroutine produced by AceGen is efficient and robust, since the code generator simplifies the symbolic expressions and has built-in optimization tools. A detailed description of AceGen features can be found e.g., in [66].

In fact, following the recommendation of Korelc [66], instead of introducing discretization into the residual Equations (39), (40) and optionally (41), pseudo-potentials are formulated for the equilibrium, energy balance and plastic strain averaging, minimization of which is equivalent to the residual equations. Specific forms of the potentials related to the governing equations considered in this model can be found in [42]. It should be mentioned that the model under consideration involves large strain plasticity and requires a solution of the non-linear set of equations at the level of Gauss points to calculate the values of internal variables. Thus, the relations between the internal variables and the global unknown fields are not given as explicit functions and the process of the automatic differentiation has to be affected by the definition of appropriate exceptions. For more details, the reader is referred to [42,66,67].

Discretization is introduced into the pseudo-potentials following the classical Ritz approach, which yields a more robust solution procedure. An extensive description of the AceGen implementation of a large strain (gradient-enhanced) thermo-plasticity models can be found in [42,43]. The codes for thermo-visco-plasticity with the McCormick extension given in Equation (23) are developed based on the same approach.

The user subroutines in AceGen are prepared for three-dimensional finite elements, in particular hexahedral elements H8 with linear interpolation of all fields (displacement, temperature and, if relevant, averaged strain) and eight Gauss points. The linear interpolation is favorable in terms of computational effort, but it is known that plasticity simulations are affected by volumetric locking if full integration is used. Therefore, the so-called F-bar enhancement, see e.g., [68], is employed for the mechanical part of the formulation.

## 5. Simulation of PLC Effect

### 5.1. Test Description

**Figure 1:**
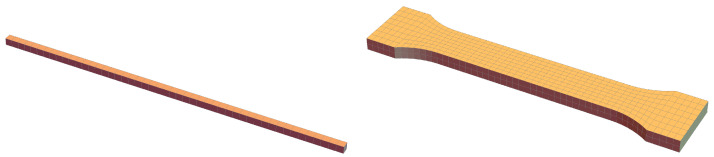
Geometry of samples and meshes, rod (**left**) and bone-shape sample (**right**).

In the numerical simulations of the PLC effect, two different samples are considered. First, an example computation for a simple tensile rod sample is made to show how the propagative instability is reproduced; see Figure 1, left. The rod dimensions are 10 × 10 × 500 mm. One end of the rod is fixed and a longitudinal displacement increasing to 75 mm (15% of the sample length) is uniformly applied at the other end within 100 s (the strain rate is 1.5 × 10${}^{-3}$ s${}^{-1}$). Fifty identical hexahedral elements with linear interpolation of all fields are used and one element is used in the cross section.

Then, simulations are performed for a configuration based on the experimental bone-shape plate sample analyzed in [49]; see Figure 1, right. The dimensions of the computed configuration are as follows: total length 102 mm, length of the middle part 57 mm, thickness 4 mm, width of middle part 12.5 mm, radius of fillets 12.5 mm, width of broader parts 20 mm. The bone-shape sample is uniformly elongated by 14.25 mm in 285 s. The element size in the central part of the mesh is approximately 2 mm.

For both specimens, insulation thermal boundary conditions are applied. The basic set of material model parameters for our study are taken from [13,25,49] for room temperature 25 °C and listed in Table 1. The results of the simulations are compared with the experimental results from [49].

The two variants of the material model described in Section 2 are used: the thermo-visco-plastic model and the thermo-visco-plastic model with the gradient enhancement. For comparison with experiments, two options for the characteristic time of solute diffusion $t_{0}$ (called solute diffusion time in brief) are considered: either constant or depending on temperature. After the comparison with experiments the parametric study is performed. For the thermo-visco-plastic model, four sets of computations are made for different values of viscosity, heat conductivity, solute diffusion time, and maximum tension time. For the gradient-enhanced model, two sets of computations are carried out for different values of internal length and ductility parameter.

**Figure 2:**
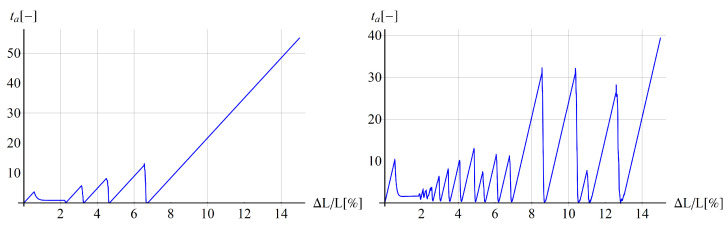
Strain ageing time vs. longitudinal displacement for rod benchmark at the right end of the sample (**left**) and for the bone-shape plate in tension at the centre of the sample (**right**).

In Figure 2, the relations between the strain aging time $t_{a}$ and the relative extension ΔL/L are plotted for the two analyzed tests and selected points in the configurations, showing abrupt jumps of $t_{a}$ according to the McCormick model, representing the DSA phenomenon. The left plot is obtained for the tensile rod, and the right one for the bone-shape sample.

To avoid convergence problems, the thermo-plastic model with the gradient enhancement is not used without the viscosity part. Since the McCormick part of the yield strength introduces recurring strain rate softening stages, the non-linear simulation algorithm fails without viscosity and it seems the gradient term is insufficient to prevent this.

### 5.2. Tensile Rod Benchmark Test

To simulate the PLC effect in a one-dimensional tension benchmark a simple 3D rod test is performed, see Figure 1 left.

**Figure 3:**
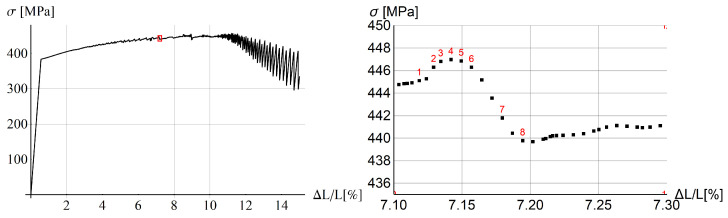
Stress vs. relative longitudinal displacement for rod benchmark: whole process (**left**) and one selected serration (**right**).

In Figure 3 stress vs. the rod extension is plotted for the whole process (left plot) and the magnification of a one serration (right plot). The place of the arbitrarily chosen serration is marked by the red box on the left plot. Small serrations are visible at the beginning of the process and they gradually grow. After the diagram peaks, the serrations grow extensively, when they actually should vanish. Obviously, the model needs some modification to prevent this kind of behavior at the final (failure) stage, but proper modeling of this stage is outside the focus of this work.

**Figure 4:**
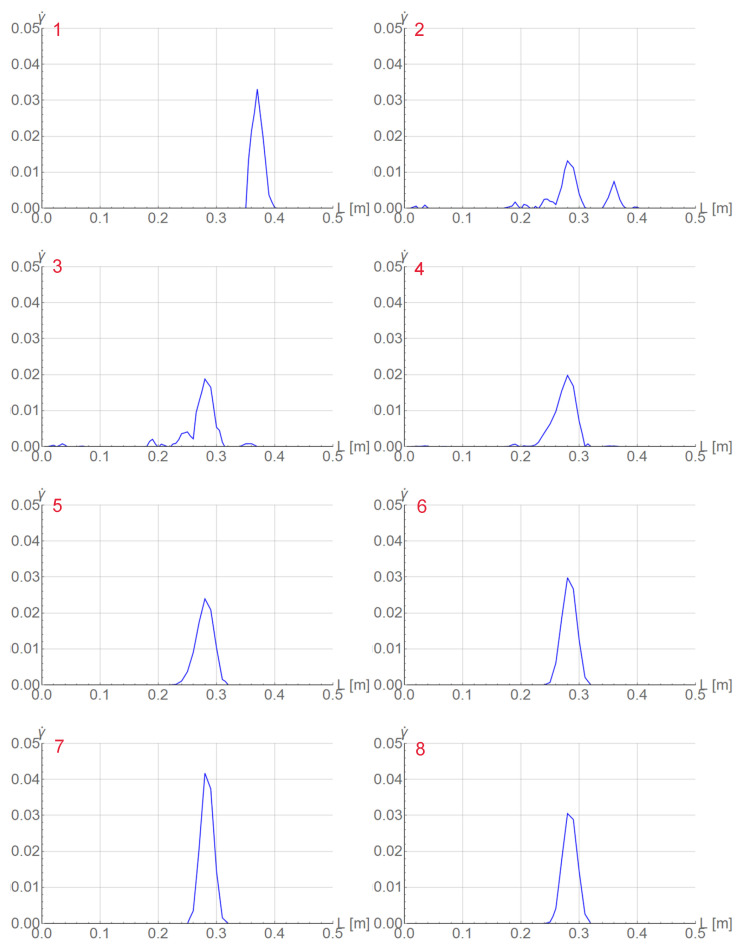
Distributions of $\dot{\gamma }$ at numbered states of selected serration for tensile rod test.

In the right diagram in Figure 3 selected states are numbered in red. In Figure 4 two columns of plots are presented for the serration and those states. Each plot shows the distribution of equivalent strain rate $\dot{\gamma }$ along the rod. For steps 2 and 3 before the peak, the band has a distributed form, while in the previous step 1, the band is localized. In the steps after the peak, $\dot{\gamma }$ localizes again at a different position.

### 5.3. Comparison with Experiments for Bone-Shape Sample

The comparisons with experiments from [49] are made for the thermo-visco-plastic model and for its gradient-enhanced version, for three initial temperatures: 25 °C, 100 °C, and 200 °C. Table 2 contains the values of the Young modulus, initial and final yield strength for the three temperatures.

**Figure 5:**
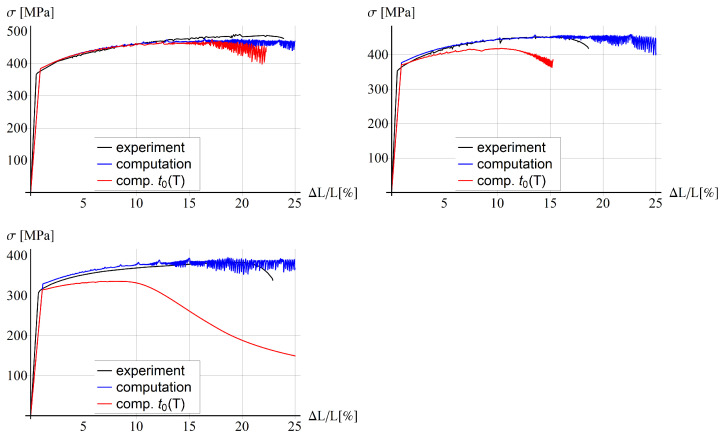
Stress vs. relative extension for thermo-visco plastic model for 25 ${}^{{}^{\circ}}$C (**left**, **top**), 100 °C (**right**, **top**), 200 °C (**left**, **bottom**).

**Figure 6:**
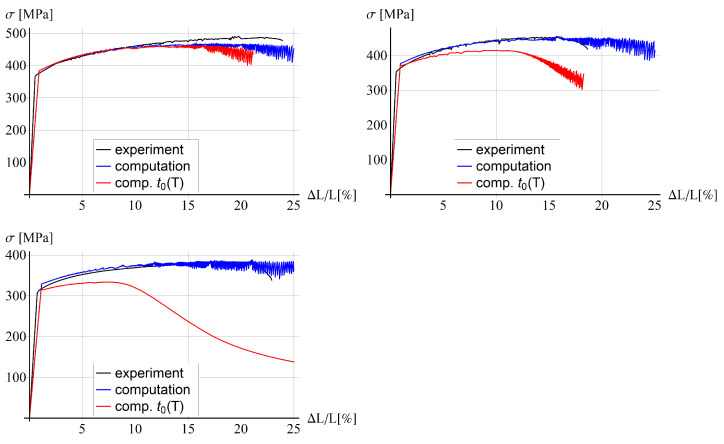
Stress vs. relative extension for thermo-visco plastic model with gradient enhacement for 25 °C (**left**, **top**), 100 °C (**right**, **top**), 200 °C (**left**, **bottom**).

Figure 5 presents the results obtained for the former model and Figure 6 for the latter one. The EMC material model parameters are fitted for the case without the dependence of the solute diffusion time $t_{0}$ on temperature. The small differences in the elastic part of the response can be caused by neglecting the elongation of the broader sample parts in the computational experiment. Notice that the level of the yielding initiation is reduced with temperature, which is related to thermal softening, and the numerical model correctly reproduces the behaviour.

When the constant value of $t_{0}=0.125$ s is assumed according to [13], we can observe in Figure 5 a good agreement in terms of global load–deformation response for the temperature equal to 25 °C and a partial agreement for higher temperatures. For 100 °C and 200 °C the blue lines are close to the experimental black lines at the beginning of the process, but they do not mimic the failure at the end for a similar extension as in the experiments. The red line for the model with the solute diffusion time depending on temperature enters softening a bit earlier for room temperature, but it shows a much softer response, far from the experimental diagrams, for higher temperatures. For the temperature equal to 100 °C and 200 °C the softening stage is entered much earlier than in the experiment. In the latter case, all serrations have been smoothed, which is similar to the findings presented in [25]. The values of model parameters $t_{01}$ and $t_{02}$, which control the dependence of $t_{0}$ on temperature, are based on [25], but obviously the exponential character of function $t_{0}\left(T\right)$ is not a suitable choice.

A similar behaviour as for the thermo-visco-plastic model can be observed for the variant with the gradient enhancement for the internal length l=5 mm and ductility parameter β=0.1, see Figure 6. It seems that the gradient enhancement of the model with the assumed material parameters, related to an additional yield strength reduction, has a minor influence on the simulated stress-relative elongation diagrams. This aspect is further analyzed in the parametric study, as follows.

### 5.4. Parametric Study

Parametric studies for the two model variants, without and with the gradient enhancement, are performed. Six parameters are taken into account. For the thermo-visco-plastic model, the following parameters are varied: viscosity ξ (5, 40, 80 MPa·s), conductivity *k* (0, 50, 121, 200 J/(s·K·m)), solute diffusion time $t_{0}$ (0.01, 0.025, 0.125, 0.5, 5, 1000 s), and the duration of the elongation process $t_{\mathrm{MAX}}$ (28.5, 285, 2850 s).

For the gradient-enhanced model, the ductility β (0.1, 0.5, 1, 2 [-]) and the internal length *l* (0, 5, 10, 20 mm) are changed. The parametric studies are carried out for the reference temperature equal to 25 °C; thus, the mechanical material parameters (Young modulus, the initial and ultimate yield strengths) are appropriate for this assumption and are taken from [49]. As given in Table 1, the default values of varied parameters are: viscosity ξ=40, conductivity k=121, and solute diffusion time $t_{0}=0.125$. Moreover, the maximum tension time $t_{\mathrm{MAX}}=285$.

#### 5.4.1. Thermo-Visco-Plastic Model

**Figure 7:**
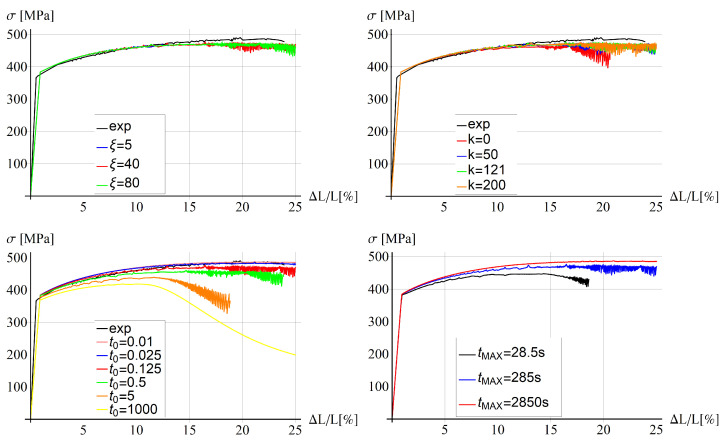
Stress vs. relative extension for different values of viscosity (**top**, **left**), conductivity (**top**, **right**), solute diffusion time (**bottom**, **left**) and maximum tension time (**bottom**, **right**).

In Figure 7, stress–strain diagrams for different values of viscosity (left, top), conductivity (right, top), solute diffusion time (left, bottom) and maximum tension time (right, bottom) are shown. The experimental diagram (black) for 25 °C is added for reference. There are no significant differences between the plots for different values of viscosity in the examined range of values; however, in the case when the viscosity ξ is equal to zero (not displayed here), the computation stops at the beginning of the process.

In the second plot, it can be observed that the response is more brittle for smaller values of heat conductivity and no significant differences can be observed for higher values of *k* than 50 J/(s·K·m). The conductivity equal to 121 J/(s·K·m) is the value characteristic for the aluminium alloy used in the experiments described in [49].

The characteristic time of the solute diffusion affects the diagram smoothness and the load-carrying capacity. For high and low values of $t_{0}$, the diagrams are smoother and there are no excessive serrations at the end of the process. The diagrams are ordered from the largest value of $t_{0}$ (the most brittle response) to the smallest value (most ductile), which means the yield strength is lower for higher values of the solute diffusion time.

The last diagram in Figure 7 shows that the response is rate dependent and the higher the load rate (the smaller $t_{max}$ is), the smaller the predicted load-carrying capacity. The diagram for the largest value of $t_{max}$ is smooth and does not exhibit serrations at the end of the process contrary to the other diagrams.

**Figure 8:**
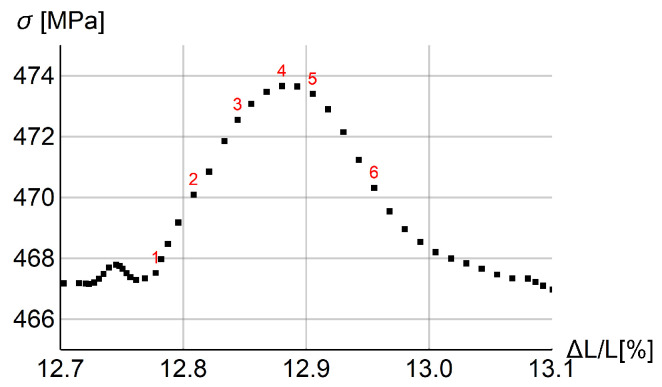
Stress vs. relative extension for one serration with step numeration (ξ=40 MPa·s, k=121 J/(s·K·m) and $t_{0}=0.125$ s).

**Figure 9:**
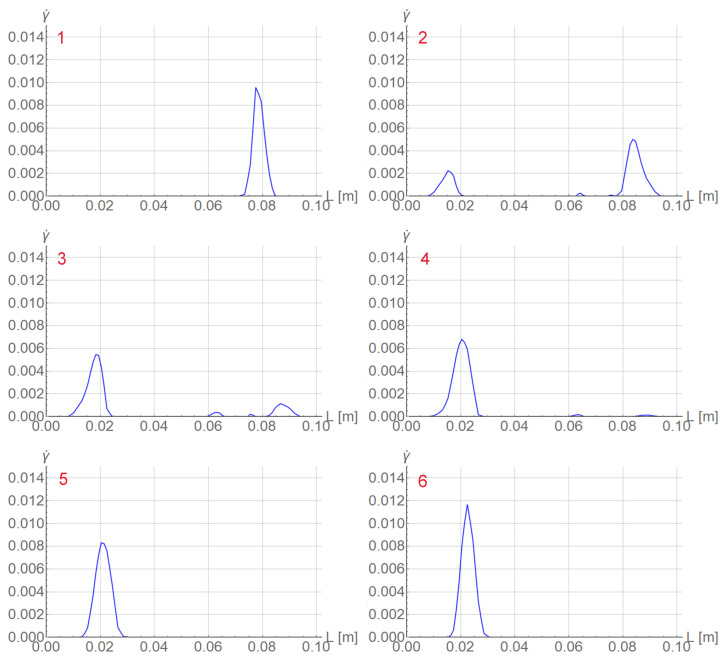
Distributions of $\dot{\gamma }$ along the specimen axis at numbered states for ξ=40 MPa·s, k=121 J/(s·K·m) and $t_{0}=0.125$ s.

**Figure 10:**
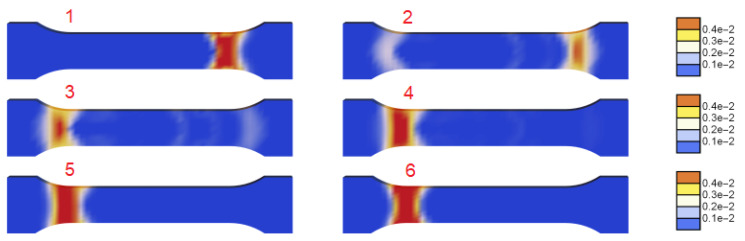
Distributions of $\dot{\gamma }$ at numbered states (ξ=40 MPa·s, k=121 J/(s·K·m) and $t_{0}=0.125$ s).

The next Figure 8 and Figure 9 are plotted for ξ=40 MPa·s, k=121 J/(s·K·m), $t_{0}=0.125$ s and $t_{max}=285$ s. In Figure 9, two columns with distributions of $\dot{\gamma }$ along the central longitudinal axis of the dog-bone sample are shown for the numbered states in the load–displacement plot part for the selected serration shown in Figure 8. In Figure 10 the distributions of $\dot{\gamma }$ in the sample are shown corresponding to the results presented in Figure 9. Before the serration peak, a band is visible on the right-hand side of the sample; see Figure 9, first row left. Then, when the peak is approached, the band on the right starts to disappear and a band on the left-hand side appears. After the peak, see Figure 9 third row left, the traces of the right band vanish completely and only the left band is visible. The same sequence of states can be observed in Figure 10.

#### 5.4.2. Thermo-Visco-Plastic Model with Gradient Enhancement

**Figure 11:**
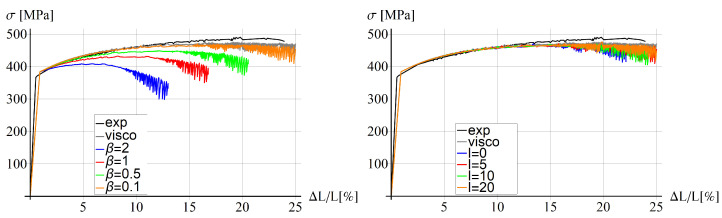
Stress vs. relative extension for different values of ductility and l=5 mm (**left**) and for different internal lengths and β=0.1 (**right**).

In Figure 11, diagrams for different values of the ductility parameter (left) and the internal length (right) are shown. For comparison, two additional diagrams are added; the black line is the experimental diagram for 25 °C, and the gray line is for the thermo-visco-plastic model with the following parameters: ξ=40 MPa·s, k=121 J/(s·K·m), $t_{0}=0.125$ s and $t_{max}=285$ s.

When the value of the ductility grows, the influence of the exponential reduction factor increases. It can be observed in Figure 11 (left) that for larger values of ductility, the load-carrying capacity is smaller and softening starts to dominate faster. The diagrams for different values of the internal length are presented in Figure 11 (right). There are no significant differences for the values of internal length larger than zero. The diagrams are close to the diagram obtained for the thermo-visco-plastic model. The diagram for l=0 (blue line ) is slightly more brittle and ends for ΔL/L≈22 due to divergence of the simulation.

**Figure 12:**
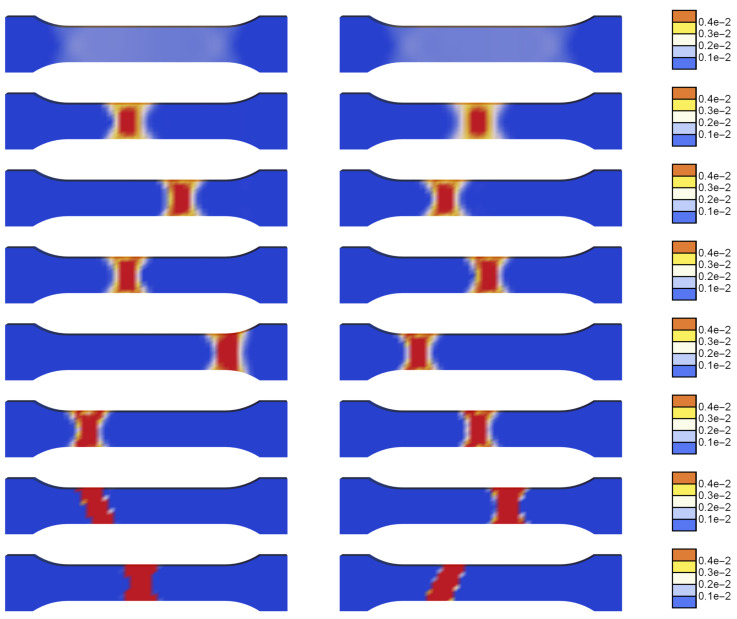
Distributions of $\dot{\gamma }$ at selected states in the deformation history for l=0 (**left column**) and l=20 mm (**right column**). For the rows of figures from top ΔL/L is equal to 3, 5, 7.5, 10, 12.5, 15, 17.5, 20.

In Figure 12, the distributions of the plastic strain rate are compared for a series of states in the deformation history. The plots on the left are for l=0 and the right ones for l=20 mm. For small deformation, a uniform distribution of $\dot{\gamma }$ is observed, then a localized band is formed, which resembles a cross pattern of shear bands diffused by regularization and/or re-hardening. The reason can also be a too-coarse finite element mesh used for the simulation. The band travels through the process zone of the sample in a similar way irrespective of the assumed internal length. The plots do not show the expected influence of the length scale on the widths of the propagating localization zones. This is probably caused by the fact that the viscosity and heat conductivity provide sufficient regularization and the gradients active on the softening parts of the serrations merely counteract the additional softening source related to the yield stress degradation involved in the gradient-enhanced model.

**Figure 13:**
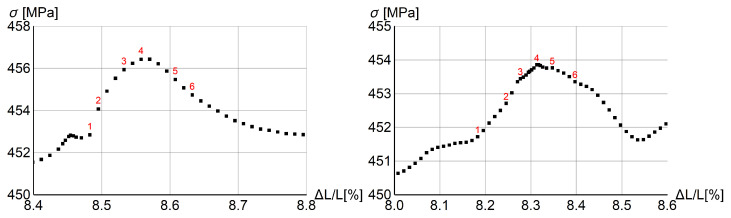
Stress vs. relative extension for selected serration and two values of internal length l=0 (**left**) and l=20 mm (**right**).

**Figure 14:**
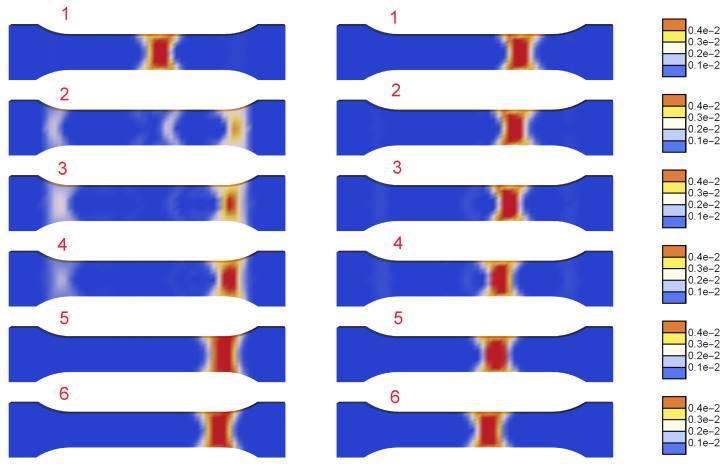
Distributions of $\dot{\gamma }$ at selected states in the deformation history, marked by numbers in Figure 13, for l=0 (**left column**) and l=20 mm (**right column**).

Figure 13 presents parts of the stress-strain diagrams of one selected serration for two values of internal length l=0 (left) and l=20 mm (right). Further, Figure 14 shows the evolution of the distribution of the plastic strain rate $\dot{\gamma }$ for the two values of the internal length scale within one selected serration presented in Figure 13. The plots for l=0 show disappearing and reappearing localization zones, while the plots for l=20 mm present a moving band. However, also for one serration the maps show a negligible influence of the length scale on the widths of the propagating bands.

## 6. Conclusions

The Portevin–Le Chatelier (PLC) effect has been simulated using two versions of the large strain thermo-plastic Estrin–McCormick model. The model is capable of reproducing the results of the Dynamic Strain Aging (DSA) phenomenon: serrations in the load–displacement diagrams (repetitive changes of softening and hardening) and propagating localization zones. The model takes into account visco-plasticity and the second version also includes a gradient enhancement via an averaging equation for the equivalent plastic strain.

Two different configurations are used in simulations. A simple rod is considered first to show how the adopted constitutive model represents the PLC phenomenon. Then, tension of the experimental bone-shape sample from [49] is simulated, instability formation and propagation are studied, and a comparison with experimental results is performed. For both tests, detailed analyses of the evolution of the plastic strain rate for a selected serration have been presented. It seems that the localization band moves, but it rather gradually disappears and then reappears at a different position.

Further, a parametric study is performed. Different values of viscosity, conductivity, the time of solute diffusion in the DSA model, and the maximum tension time (loading rate) have been considered for the thermo-visco-plastic model. For the model with the gradient enhancement, the ductility parameter and the internal length have varied.

On one hand, the results of simulations are quite satisfactory: the simulated load–extension diagrams are quite close to the experimental results and the sensitivity to model parameters is in agreement with expectations. On the other hand, excessive post-peak serrations are visible for most of the computation, so a method to reduce them is needed. The adopted dependence of the parameters of the McCormick model on temperature led to results far from the experimental ones for higher temperatures, so this aspect requires further research and model improvement. Moreover, experimental studies are necessary to compare the shear band evolution in PLC simulations (and not only load–displacement plots) and to identify material model parameters in a similar way to [13,25].

Finally, the distributions of the equivalent plastic strain rate in the specimen for the gradient-enhanced model are examined for two values of the internal length scale l=0 and l=20 mm. They are compared for a series of states showing no significant differences in the size of the localization bands. This can be caused by the relatively coarse discretization used, or by the fact that viscosity and heat conduction provide some regularization, which manifests itself in smoothing of the simulated bands and in a weak influence of gradients.

## Figures and Tables

**Table 1:** Basic set of mechanical, thermal and McCormick model parameters.

Property	Symbol	Value	Unit
Young modulus	*E*	68.56	GPa
Poisson ratio	ν	0.3	-
Initial yield strength	$\sigma_{y0}$	367.5	MPa
Final yield strength	$\sigma_{yf}$	488.8	MPa
Saturation constant	δ	16	MPa
Viscosity	ξ	40	MPa · s
Conductivity	*k*	121	J/(s·K·m)
Heat capacity	*c*	2,423,750	J/($m^{3}\cdot^{{}^{\circ}}$C)
Thermal expansion coeff.	$\alpha_{T}$	23.2 $\times \;10^{-6}$	1/°C
Thermal softening modulus	$H_{T}$	0.0016	1/°C
Heat dissipation factor	χ	0.9	-
Solute diffusion time	$t_{0}$	0.125	s
EMC model param.	$\Omega_{0}$	13.62 × 10${}^{-4}$	-
EMC model param.	$\Omega_{0}^{\prime }$	7.2 × 10${}^{-4}$	-
EMC model param.	$\sigma_{B00}$	18.9	MPa
EMC model param.	$\sigma_{B00}^{\prime }$	567.78	MPa
EMC model exponent	*n*	$3^{-1}$	-
EMC model param.	$t_{01}$	0.051355	1/°C
EMC model param.	$t_{02}$	0.03462	s

**Table 2:** Parameters for different temperatures.

Property	Symbol	25 °C	100 °C	200 °C	Unit
Young modulus	*E*	68.56	65.56	46.62	GPa
Initial yield strength	$\sigma_{y0}$	367.5	360.3	312.9	MPa
Final yield strength	$\sigma_{yf}$	488.8	466.2	383.2	MPa

## Data Availability

The data presented in this study are available in the article.

## Abbreviations

The following abbreviations are used in this manuscript:

EMC	Estrin–McCormick model.
PLC	Portevin–Le Chatelier.
DSA	Dynamic Strain Aging.
HMH	Huber–Mises–Hencky.
BVP	Boundary Value Problem.
FE	Finite Elements.
